# Co-occurring autism, ADHD, and gender dysphoria in children, adolescents, and young adults with eating disorders: an examination of pre- vs. post-COVID pandemic outbreak trends with real-time electronic health record data

**DOI:** 10.3389/fpsyt.2024.1402312

**Published:** 2024-08-20

**Authors:** Tashalee R. Brown, Madeline O. Jansen, A. Ning Zhou, Dominic Moog, Hui Xie, Katherine V. Liebesny, Kevin Y. Xu, Binx Y. Lin, Wisteria Y. Deng

**Affiliations:** ^1^ Department of Psychiatry and Biobehavioral Sciences, David Geffen School of Medicine at University of California-Los Angeles (UCLA), Los Angeles, CA, United States; ^2^ Jane and Terry Semel Institute for Neuroscience and Human Behavior at University of California-Los Angeles (UCLA), Los Angeles, CA, United States; ^3^ San Francisco Department of Public Health, San Francisco, CA, United States; ^4^ Department of Psychiatry and Behavioral Sciences, University of California, San Francisco, CA, United States; ^5^ Washington University School of Medicine, St. Louis, MO, United States; ^6^ Zilber School of Public Health, University of Wisconsin-Milwaukee, Milwaukee, WI, United States; ^7^ Department of Psychiatry and Behavioral Medicine, Carilion Clinic - Virginia Tech Carilion School of Medicine, Roanoke, VA, United States; ^8^ School of Medicine, Washington University in St. Louis, St. Louis, MO, United States; ^9^ Department of Psychiatry, Division of Addiction Science, Prevention and Treatment, Washington University School of Medicine, St. Louis, MO, United States; ^10^ Department of Psychology, Yale University, New Haven, CT, United States

**Keywords:** eating disorder, autism, ADHD, gender dysphoria, children, adolescent and young adult, real-world data

## Abstract

**Background:**

Incidence rates of autism, attention-deficit/hyperactivity disorder (ADHD), and gender dysphoria (GD) are rising not only in the general population, but particularly among children, adolescents, and young adults with eating disorders (EDs). While ED rates have risen during the COVID pandemic, trends in co-occurring autism, ADHD, and GD have yet to be investigated in detail or at scale by way of large electronic medical record data.

**Objectives:**

To investigate trends in rates of co-occurring autism, ADHD, and GD among children, adolescents, and young adults with EDs in years prior to and during the COVID-19 pandemic.

**Methods:**

We utilized a de-identified multinational electronic health records database (TriNetX) with 48,558 individuals aged 5-26 diagnosed with eating disorders (EDs) at least twice between 2017 and 2022. The primary predictor variable differentiated between the years of each person’s index (first) ED diagnosis (2017-2019 vs. 2020-2022). The primary outcome variable was the rate of new co-occurring psychiatric diagnoses of autism, ADHD, and GD in the year following each patient’s first ED diagnosis. We applied propensity score-matched multivariable logistic regressions to compare primary outcomes between 2017-2019 and 2020-2022.

**Results:**

Our analysis included 17,445 individuals diagnosed with EDs in 2017-2019 (8% autism, 13.5% ADHD, 1.9% GD) and 31,113 diagnosed with EDs in 2020-2022 (8% autism, 14.6% ADHD, 3.2% GD). After 1:1 propensity score matching, 17,202 individuals from the 2017-2019 cohort were matched to peers mirroring the 2020-2022 cohort. Those diagnosed in 2020-2022 showed a 19% (aOR[95%CI]=1.19[1.07-1.33]), 25% (aOR=1.25[1.04-1.49]), and 36% (aOR=1.36[1.07-1.74]) increase in odds for autism, ADHD, and GD diagnoses, respectively, within the 365 days after the index EDs diagnosis, compared to the 2017-2019 cohort.

**Discussion:**

Rates of autism, ADHD, and GD are significantly higher in individuals with ED in the post-pandemic 2020-2022 cohort in comparison to the pre-pandemic 2017-2019 cohort, even after controlling for baseline levels of co-occurring psychiatric diagnoses. Such findings reveal a critical gap in our current understanding of the totality of ways in which COVID-19 may have impacted the onset and clinical course of EDs, autism, ADHD, and GD among children, adolescents, and young adults.

## Introduction

Eating disorders (EDs) have been increasing in incidence in the United States (U.S.) since the beginning of the COVID-19 pandemic (1), with resultant prevalence and severity posing an unprecedented impact upon those affected (2, 3). Unfortunately, there has been a dearth of research examining the risk of co-occurring psychiatric conditions among individuals affected by EDs. This is an important research gap because prior studies suggest that nearly half of all individuals with EDs also have co-occurring psychiatric diagnoses (4). Furthermore, the successful treatment of EDs often requires diligent attention to the screening, diagnosis, evaluation, and treatment of co-occurring psychiatric conditions (5). Lastly, co-occurring psychiatric conditions in those with EDs often correspond with increased ED symptom burden and worse outcomes (6, 7).

The high prevalence of co-occurring depressive-, anxiety-, and obsessive-compulsive disorder-spectrum illnesses in individuals with EDs is well-known and well-documented in the scientific literature to date (8, 9). Yet, data over the last 10 years has shown that EDs also highly co-occur with autism and attention-deficit/hyperactivity disorder (ADHD). In particular, autism and ADHD have historically been overlooked in existing ED literature. In nationally representative samples in the U.S., ADHD was found to be robustly associated with EDs, both in lifetime and across past 12-month analyses (10). In fact, one five-year prospective study found that girls with ADHD were nearly four-times more likely to develop EDs than peers without ADHD (11). Recent reports have highlighted that the prevalence of autism is significantly higher in those with EDs, compared to the general population (12–14). Studies also indicated the overlapping traits between autism and ED at clinical and potentially mechanistic levels (13, 15–17)For example, repeating patterns of behavior shared in autism and ADHD might be the bridge to disordered eating. Furthermore, studies have shown that greater severity of co-occuring ADHD symptoms correspond to higher rates of ED treatment discontinuation (18). The severity of ED symptoms may also be exacerbated by co-occurring autism (19) and GD (20).

While EDs have been historically posited to affect cisgender women (i.e. those whose gender aligns with their assigned sex at birth) more than cisgender men (21), such statements fall short in recognizing the needs of gender diverse individuals (i.e. those whose identified gender does not align with their assigned sex at birth). Indeed, recent research has raised concerns about the rising rates of EDs among gender diverse cohorts (22–24). These findings highlighting a high co-occurrence between EDs and gender diversity are further supported by recent work indicating significantly higher rates of EDs in gender non-binary youth (i.e. those whose gender identity is outside the male/female binary) compared to cisgender peers, with some studies estimating the prevalence rate of ED as high as 18% in transgender youth (22). The increased risk of EDs in gender diverse individuals is particularly multifactorial and complex; for example, ED behaviors may be associated with body dissatisfaction or serve to alter physical features to be more in line with an individual’s gender identity (23, 24).

To our knowledge, there is little research that uses large real-world data to evaluate trends in the diagnoses of co-occurring psychiatric conditions among gender diverse individuals with EDs during the COVID-19 pandemic, even as research has shown significant increases in the youth diagnosed with autism (25), ADHD (26), and gender dysphoria (GD) (27) in the U.S. Procuring descriptive data on trends in co-occurring psychiatric diagnoses in people with EDs represents a crucial first step towards developing targeted interventions to improve treatment outcomes in gender diverse individuals with EDs, especially in the post COVID-19 era. The present study seeks to use a multi-national real-time administrative claims database for over 118 million enrollees (private, public insurance) to evaluate changes in the prevalence of co-occurring psychiatric diagnoses in people with EDs, with a focus on autism, ADHD, and GD. Such changes will be evaluated across timepoints prior to and following the COVID-19 Pandemic in order to assess its impact upon diagnostic trends among an U.S. Youth population.

## Methods

### Overview

The TriNetX databases are a federated multinational research network that captures de-identified real-time electronic health records from inpatient and outpatient settings, covering over 118 million people across 84 healthcare organizations (>90% U.S. based) (28). The data, while rich, lacks detailed geographic (i.e., patient zip codes and addresses) and institutional (i.e., provider and setting type) information for the purposes of safeguarding real-time protected health information. Detailed information on the TriNetX data’s privacy-preserving procedures for enriching clinical EHR data with closed claims and institutional mortality have been previously described (28). The TriNetX data uses privacy-preserving record linkage procedures to create a longitudinal EHR dataset that avoids counting individuals multiple times. Given the use of deidentified secondary data, our analysis was determined to be non-human subjects research from the IRB review at Carillon Clinic.

### Population and variables

Our population comprised of individuals aged 5-26 years diagnosed with eating disorders (ICD-10-CM: F50). The *predictor variable* was pre- versus post-COVID-19 outbreak timing of ED diagnosis, using January 1, 2020 as the threshold separating the two time periods. Two cohorts were curated: (1) individuals receiving ED diagnoses at least twice [to improve the validity of ICD diagnoses (29)] between January 1, 2017 and December 31, 2019, and (2) individuals receiving ED diagnoses at least twice between January 1, 2020 and December 31, 2022. The *outcome variable* was the diagnosis of co-occurring psychiatric conditions from day 0 to day 365 following the index event (first ED diagnosis within the timeframe).

To focus on active psychiatric conditions that were present at the time of first ED diagnosis and later, we excluded prior outcome measures (aka. historical psychiatric diagnoses or conditions in remission) preceding the new ED diagnoses. Baseline covariates in the propensity score matching analysis included demographics (age, race and ethnicity, gender) and the diagnosis of co-occurring psychiatric conditions in the one year *prior to* the first ED diagnosis (i.e., autism, ADHD, GD, generalized anxiety disorder, mood disorders such as depression and bipolar disorder, substance use disorders, obsessive-compulsive disorder). ICD-10-CM codes are detailed in the online supplement.

### Statistical analysis

Descriptive analyses were conducted using HIPAA-compliant analytical tools that are built into the TriNetX interface. Propensity scores have been employed to match people pre-pandemic (2017-2019) with similar individuals during the pandemic on sociodemographic (i.e., age, sex, ethnicity, race) and clinical characteristics (i.e., mood disorders, anxiety-spectrum disorders, substance use disorders) in an effort to mitigate confounding by indication (9, 30, 31). A full listing of variables used for propensity score estimation is shown in **Table 1**, where covariate balance is assessed via absolute standardized differences. We used the greedy nearest neighbor matching with a caliper of 0.1 pooled standard deviations. Adjusted multivariable logistic regressions were conducted to estimate the adjusted odds ratio (aOR) and 95% confidence intervals (95%CIs) of co-occurring psychiatric diagnoses between the pre- and post-2020 cohorts.

**Table 1:** Demographic and clinical characteristics of cohorts 1 year before index event.

Variables	Before Matching						After Matching					
	Patients in 2020-2022	%	Patients in 2017-2019	%	P-Value	Std diff.	Patients in 2020-2022	%	Patients in 2017-2019	%	P-Value	Std diff.
N	31,113	100.0%	17,445	100.0%			17,204	100.0%	17,204	100.0%	<0.001	0.60
Current Age	17.9 ± 4.7	–	19.4 ± 4.9	–	<0.001	0.31	16.7 ± 4.8	–	19.6 ± 4.7	–	<0.001	0.60
Age at Index	15.3 ± 4.7	–	13.9 ± 4.9	–	<0.001	0.30	14.1 ± 4.8	–	14.0 ± 4.7	–	0.48	0.01
Sex												
Female	24,481	78.7%	12,951	74.2%	<0.001	0.11	12,855	74.7%	12,896	75.0%	0.61	0.01
Male	6,227	20.0%	4,396	25.2%	<0.001	0.12	4,251	24.7%	4,210	24.5%	0.61	0.01
Unknown	405	1.3%	98	0.6%	<0.001	0.08	98	0.6%	98	0.6%	0.00	1.00
Ethnicity												
Not Hispanic or Latino	22,026	70.8%	12,890	73.9%	<0.001	0.07	12,755	74.1%	12,677	73.7%	0.34	0.01
Unknown Ethnicity	5,286	17.0%	2,354	13.5%	<0.001	0.10	2,354	13.7%	2,345	13.6%	0.89	0.00
Hispanic or Latino	3,801	12.2%	2,201	12.6%	0.20	0.01	2,095	12.2%	2,182	12.7%	0.16	0.02
Race												
White	20,989	67.5%	11,839	67.9%	0.36	0.01	11,786	68.5%	11,699	68.0%	0.31	0.01
Black or African American	2,287	7.4%	1,463	8.4%	<0.001	0.04	1,351	7.9%	1,406	8.2%	0.28	0.01
Other Race	1,756	5.6%	1,040	6.0%	0.15	0.01	989	5.7%	1030	6.0%	0.35	0.01
Asian	998	3.2%	560	3.2%	0.99	<0.001	539	3.1%	548	3.2%	0.78	0.00
**Psychiatric disorders**	31,113	100.0%	17,445	100.0%	--	--	17,204	100.0%	17,204	100.0%	--	--
Mood [affective] disorders	14,307	46.0%	7,038	40.3%	<0.001	0.11	6,965	40.5%	7,037	40.9%	0.43	0.01
GAD	7,571	24.3%	3,333	19.1%	<0.001	0.13	3,299	19.2%	3,333	19.4%	0.64	0.01
OCD	1,968	6.3%	967	5.5%	0.001	0.03	888	5.2%	967	5.6%	0.06	0.02
Panic disorder	1672	5.4%	754	4.3%	<0.001	0.05	740	4.3%	754	4.4%	0.71	0.00
Specific phobia	2065	6.6%	1079	6.2%	0.05	0.02	1,017	5.9%	1,079	6.3%	0.16	0.02
PTSD	2700	8.7%	990	5.7%	<0.001	0.12	939	5.5%	990	5.8%	0.23	0.01
Adjustment disorders	1395	4.5%	672	3.9%	0.001	0.03	627	3.6%	672	3.9%	0.20	0.01
Personality disorders	1,071	3.4%	467	2.7%	<0.001	0.04	389	2.3%	466	2.7%	0.01	0.03
Borderline personality disorder	856	2.8%	357	2.0%	<0.001	0.05	316	1.8%	357	2.1%	0.11	0.02
Gender dysphoria	989	3.2%	329	1.9%	<0.001	0.08	313	1.8%	329	1.9%	0.52	0.01
ADHD	4,551	14.6%	2,352	13.5%	0.001	0.03	2,281	13.3%	2,350	13.7%	0.28	0.01
Autism	2514	8.1%	1,514	8.7%	0.02	0.02	1,497	8.7%	1,514	8.8%	0.75	0.00
Substance use disorder	2298	7.4%	946	5.4%	<0.001	0.08	849	4.9%	944	5.5%	0.02	0.03
Cannabis use disorder	1375	4.4%	599	3.4%	<0.001	0.05	535	3.1%	599	3.5%	0.05	0.02
Alcohol use disorder	474	1.5%	224	1.3%	0.03	0.02	201	1.2%	224	1.3%	0.26	0.01
Opioid use disorder	117	0.4%	56	0.3%	0.33	0.01	40	0.2%	54	0.3%	0.15	0.02
Nicotine use disorder	813	2.6%	246	1.4%	<0.001	0.09	218	1.3%	246	1.4%	0.19	0.01
Other stimulant use disorders	110	0.4%	53	0.3%	0.36	0.01	39	0.2%	53	0.3%	0.14	0.02

The STROBE (Strengthening the Reporting of Observational Studies in Epidemiology) and RECORD-PE (Reporting of Studies Conducted Using Observational Routinely Collected Health Data Statement for Pharmacoepidemiology) guidelines were followed. The data in the manuscript was queried on January 8, 2024. We used 2-sided *P* values with a <.05 threshold for statistical significance.

## Results

### Univariate and descriptive analyses

**Table 1** represents a total of 17,445 individuals with ED diagnoses in 2017-2019 and 31,113 individuals with ED diagnoses in 2020-2022 in this study. The mean age at the time of index (first) ED diagnosis was 13.9 years (Standard Deviation [SD]=4.9) in the 2017-2019 cohort and 15.3 years (SD=4.7) in the 2020-2022 cohort. 74.2% (n=12,951, 2017-2019) and 78.7% (n=24,481, 2020-2022) were female. Approximately 68% in both cohorts (n=11,839/17,445, 2017-2019 and n=20,989/31,113, 2020-2022 respectively) were White (Hispanic or non-Hispanic).

Overall, the diagnosis of co-occurring psychiatric conditions within the 365 days prior to an ED diagnosis was prevalent. Of the most common co-occurring conditions, over 40% in both cohorts (n=7,038/17,445, 2017-2019 and 14,307/31,113, 2020-2022) had diagnoses of mood disorders (i.e., major depressive disorder, bipolar disorder), and approximately 20% had diagnoses for generalized anxiety disorder (n=3,333/17,445, 2017-2019 and n=7,571/31,113, 2020-2022). The third most common disorder was ADHD, with 13.5% in the 2017-2019 cohort (n=2,352/31,113) and 14.6% in the 2020-2022 cohort (n=4,551/31,113). Autism was also relatively common, with over 8% for both cohorts (n=1,514/17,445 in the 2017-2019 cohort and n=2,514/31,113 in the 2020-2022 cohort).

While 1.9% of individuals with EDs received a diagnosis of GD in the 2017-2019 cohort (n=329/17,445), this figure was almost doubled in the 2020-2022 cohort (3.2%; n=989/31,113). Substance use disorder diagnoses increased from 5.4% (n=946/17,445) in the 2017-2019 cohort to 7.4% (n=2,298/31,113) in the 2020-2022 cohort, with cannabis use disorder being the most common substance use disorder (3.4%, n=599/17,445 and 4.4%, 1375/31,113, respectively). Only about 1% of the sample had co-occurring alcohol use disorder (1.3%, 224/17,445 in the 2017-2020 cohort; 1.5%, 474/31,113, in the 2020-2022 cohort). Whereas 1.4% (246/17,445) of the 2017-2019 cohort had diagnoses of nicotine use disorders, this figure was 2.6% (813/31,113) in the 2020-2022 cohort.

### Multivariable analyses

After propensity score matching, 17,202 individuals with EDs in the 2017-2019 cohort were matched to peers with the same demographic and psychiatric diagnostic profile as peers in the 2020-2022 cohort. With an absolute standardized differences threshold of 0.1 indicating covariate balance between groups (32), **Table 2** shows that after matching, the 2020-2022 cohort exhibited significantly elevated rates of autism, ADHD, and GD compared to peers in the 2017-2019 cohort. Specifically, individuals with EDs in the 2020-2022 cohort showed a 36% (aOR=1.36[1.07-1.74]) increased odds of having new diagnoses of GD in the 365 days following their first EDs diagnosis. Likewise, individuals in the 2020-2022 cohort showed a 19% (aOR=1.19[1.07-1.33]) and 25% (aOR=1.25[1.04-1.49]) increase in odds of ADHD and autism diagnoses in the 365 days following the index EDs diagnosis, compared to peers in the 2017-2019 cohort.

**Table 2:** Comparison of Co-occurring Psychiatric Conditions365 days following the index diagnoses with 365-day washout (2020-2022 vs. 2017-2019).

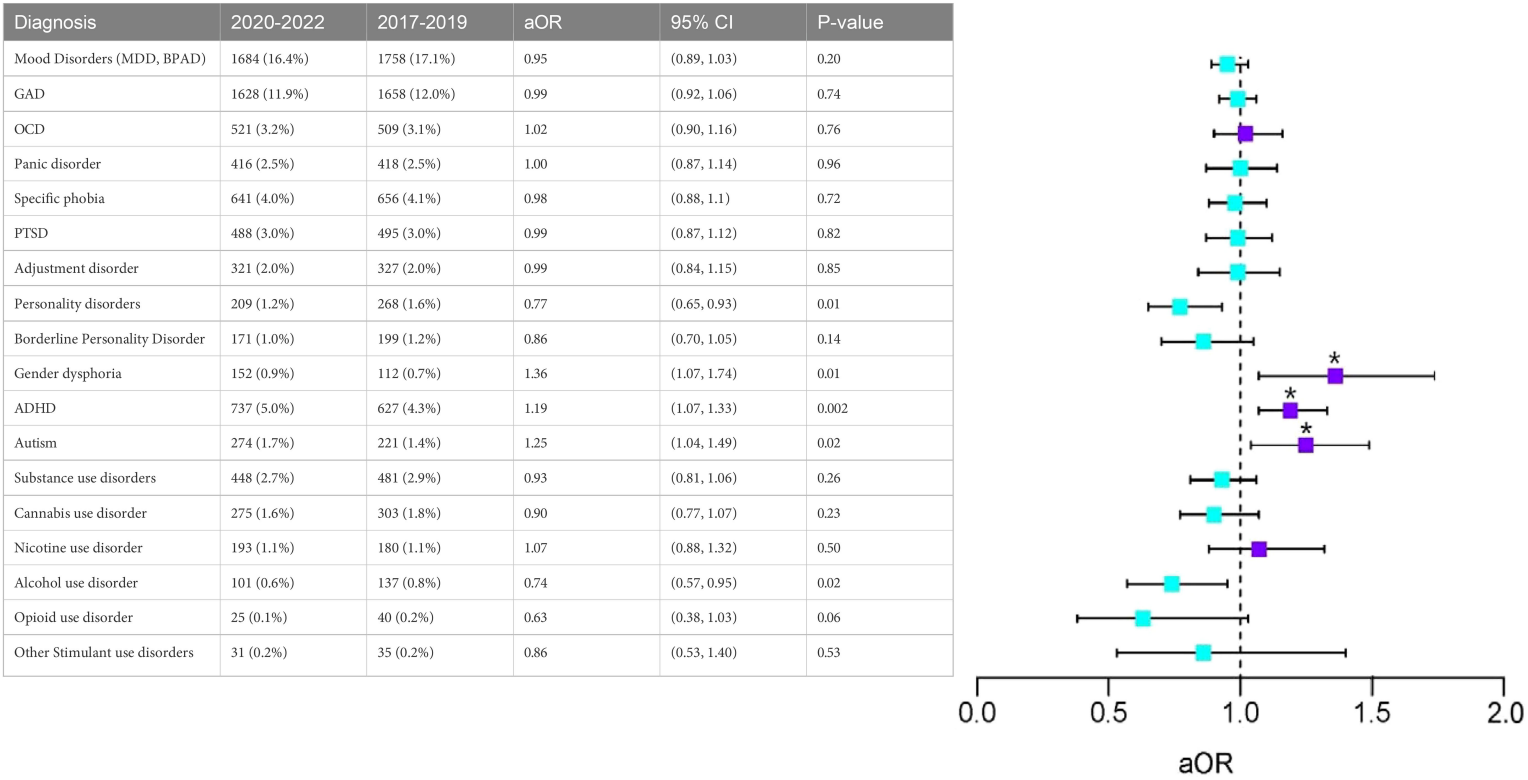

## Discussion

This analysis uses multinational real-world data to characterize trends in the prevalence of co-occurring psychiatric conditions in children, adolescents, and young adults with EDs. Prior research has suggested that rates of autism, ADHD, and GD diagnoses are rising across general U.S. population since the onset of COVID-19. While autism, ADHD, and GD are thought to be particularly prevalent in those with EDs, there is little research examining related diagnostic trends since the start of the pandemic. We thus identified matched counterparts between individuals with EDs in 2017-2019 and those in 2020-2022 with regard to similarities in psychiatric diagnostic profiles prior to receiving an ED diagnosis. Overall, our results suggest that rates of autism, ADHD, and GD are significantly higher in children, adolescents, and young adults with ED diagnoses in the pandemic 2020-2022 cohort compared to those in the pre-pandemic 2017-2019 cohort. Such findings remain consistent even after controlling for baseline levels of psychiatric diagnoses.

Reasons underlying these findings are likely to be complex and therefore warrant further dedicated investigation. For example, social distancing restrictions reduced healthcare access for many people with EDs, and many individuals experienced a significant worsening of ED, mood, and anxiety symptoms during the pandemic (33, 34). Yet, this data suggests that children, adolescents, and young adults diagnosed with autism, ADHD, and GD may have been disproportionately impacted by the pandemic. It is well known that the lockdown and home isolation contributed to major changes in the lifestyles of youth and their parents, with marked changes to daily routines, complicated by limited physical space (35), and challenges with returning to in-person schooling after extended time at home, all of which may have contributed to increased identification of autistic traits among those who were previously undiagnosed. For children who already had an autism diagnosis, the pandemic caused disruptions to therapy and education (36), as well as to support networks for caregivers (35). Likewise, for those with undiagnosed ADHD, COVID-19 often led to significant challenges in online learning, motivation, decreased access to physical activity and social outlets, and worsening externalizing behaviors, all of which may have contributed to an uptick in diagnosis (37). Furthermore, the COVID-19 crisis contributed to decreased (38) and interrupted access to gender affirming care (39) for transgender and gender diverse individuals, as well as reduced access to in-person gender affirming spaces such as school- and community-based support and advocacy groups. Gender diverse youth in unsupportive home environments may have experienced more identity-related stress and familial conflict, while those in supportive home environments may have had increased opportunities to explore and consolidate their gender identity, leading to increased recognition of the need for gender affirming medical care. Finally, social media use (i.e., TikTok) may also be playing a role in such trends, leading to more awareness of autism and ADHD, as well as higher visibility of gender diverse populations, as adolescents and young adults seek mutual support online during social distancing and lockdown (40–42).

Our work adds unique value to the existing research in several ways. First, our study covers a wide age range with samples from children and adolescents to young adults. Prior work in this area has mostly examined narrower age ranges, and there is a concerning lack of data on ED dynamics across the life span (43). While it is helpful to evaluate ED prevalence across developmental stages separately, it can be extremely informative to build a more holistic understanding of the overarching trends across psychosocial development. Such a granular approach allows for the identification of diagnostic patterns and capturing risk trajectories. Second, our study is strengthened by its attention to gender diverse youth. The association between GD and neurodevelopmental disorders (NDDs), such as autism and ADHD, has been a topic of interest, along with the possible stigma such co-occurrence incurs for gender diverse individuals. NDDs have been found to be higher in transgender and gender diverse individuals compared to their cisgender peers (25, 44). We thus hope to draw attention to an emerging public health struggle as many states fail to meet the needs of gender diverse individuals, especially in the presence of co-occurring NDDs and EDs among this population. While NDDs and GD may precede the diagnosis of ED, our study captures the increasing emergence of novel autism, ADHD, and GD diagnoses among individuals seeking ED treatment during the COVID pandemic. This suggests the presence of specific populations for whom changes in eating behaviors may be signs of undiagnosed NDDs or GD. Future work is encouraged to consider the different subjective experiences and clinical presentations of NDDs and EDs for gender diverse individuals. For example, gender diverse individuals may exhibit disordered eating behaviors to alter their gender expression (e.g., eating more to increase/decrease curves or look more feminine/masculine). Gender-diverse individuals with NDDs may face greater challenges when seeking gender affirming care, such as the threat of clinicians erroneously attributing gender diversity to a manifestation of NDDs rather than GD due to lack of awareness, training, and experience delivering gender affirming care for youth with NDDs (45). Both clinicians and researchers are urged to increase their awareness of the heterogeneous clinical presentations and diverse clinical needs, especially for patients ranging from children to young adults. The presence of EDs and NDDs in gender diverse people should not preclude access to medically necessary gender affirming care.

There are several important limitations to consider. First, we cannot rule out misclassification bias in the analyses of administrative data. While we infer that the first episode of treatment marked by an ED diagnosis represents the first ED diagnosis, it is plausible that patients may have received diagnoses earlier in time but were not captured in the database. To avoid further misclassification, we did not separate the subtypes of ED. Thus, the current study might not be best fitting to elucidate the heterogeneity of ED presentations. Similarly, the diagnoses of autism and ADHD may be at risk of misclassification in the presence of depression and anxiety (46, 47). Second, the TriNetX data only permits retrospective analyses of an observational cohort, limiting our ability to infer causation. Further, the TriNetX dataset lacks refinement capabilities due to its built-in interface, intended to protect protected health information. For instance, whereas studies have found that many patients with EDs struggled with a heightened burden of posttraumatic symptoms during the COVID-19 pandemic compared to their peers (48), detailed data on psychological vulnerabilities cannot be measured via an administrative dataset. Third, the individuals diagnosed with GD likely did not represent all the gender diverse people in this dataset. Fourth, despite our attempt to use multivariable adjustments, residual confounding is likely, and this data should be viewed as preliminary steps for in-depth future analyses. Fifth, while we are unable to restrict our data analysis to specific countries, >90% of the TriNetX data comes from the USA. We would still like to acknowledge the potentially heterogenous experience of COVID during the assessed period, given the differential public health policies and social norms by region or states. Further, our analyses may not capture data from racially minoritized communities who do not have access to the health care required for inclusion in an administrative dataset. Further, our analyses may not capture data from racially minoritized communities who do not have access to the health care required for inclusion in an administrative dataset. TriNetX only captures individuals seeking treatment and we also excluded those who had NDDs and GD before their ED diagnosis, as such, our findings may not be generalizable to those without access to care amid the myriad of barriers to healthcare access or a larger population. That said, the present study is strengthened by its unique ability to provide real-time population-level estimates.

## Conclusions

In summary, this retrospective cohort study examined a large de-identified multinational database of real-time electronic health records. The present analysis represents one of the largest and most comprehensive studies of co-occurring psychiatric diagnoses in individuals with EDs to date, depicting an elevated incidence of autism, ADHD, and GD in children, adolescents, and young adults diagnosed with ED in the pandemic era.

## Data Availability

The data analyzed in this study is subject to the following licenses/restrictions: This administrative claim data is contracted with our institution for confidentiality. Requests to access these datasets should be directed to Martha Tenzer mmtenzer@carilionclinic.org.

## Supplementary material

Supplementary Information We conducted propensity score-matched analyses that evaluate the risk of psychiatric diagnoses in the 365 days AFTER the index ED diagnosis. To accomplish this, people with EDs (above) and peers without EDs (below) are MATCHED with demographics and prior history of psychiatric diagnoses in the 365 days preceding the index date.
