# Supplementary material for: Co-occurring autism, ADHD, and gender dysphoria in children, adolescents, and young adults with eating disorders: an examination of pre- vs. post-COVID pandemic outbreak trends with real-time electronic health record data
Source: Front Psychiatry. 2024 Aug 20;15:1402312. doi: 10.3389/fpsyt.2024.1402312 (PMC11370642; doi:10.3389/fpsyt.2024.1402312)
Supplement: Supplementary file 1 [file Table1.pdf]

Supplementary Table – Coding of Variables in the TriNetX Databases

**Variables**	**ICD-10-CM codes**
Psychiatric disorders	F01-F99
Alcohol use disorder	F10
Substance use disorder	F10-19
Opioid use disorder	F11
Cannabis use disorder	F12
Other Stimulant use disorders	F15
Nicotine use disorder	F17
Mood Disorders (MDD, BPAD)	F30-F39
specific phobia	F40
Panic disorder	F41.0
GAD	F41.1
OCD	F42
PTSD	F43.1
Adjustment disorder	F43.2
Eating disorders	F50
Personality disorders	F60
Borderline Personality Disorder	F60.3
Gender dysphoria	F64
Autism	F84.0
ADHD	F90
