# Supplementary material for: Co-occurring autism, ADHD, and gender dysphoria in children, adolescents, and young adults with eating disorders: an examination of pre- vs. post-COVID pandemic outbreak trends with real-time electronic health record data
Source: Front Psychiatry. 2024 Aug 20;15:1402312. doi: 10.3389/fpsyt.2024.1402312 (PMC11370642; doi:10.3389/fpsyt.2024.1402312)

Supplementary Information

We conducted propensity score-matched analyses that evaluate the risk of psychiatric diagnoses in the 365 days AFTER the index ED diagnosis. To accomplish this, people with EDs in the 2017-2019 and 2020-2022 cohorts were MATCHED to each other via demographics and prior history of psychiatric comorbidities in the 365 days preceding the index date.

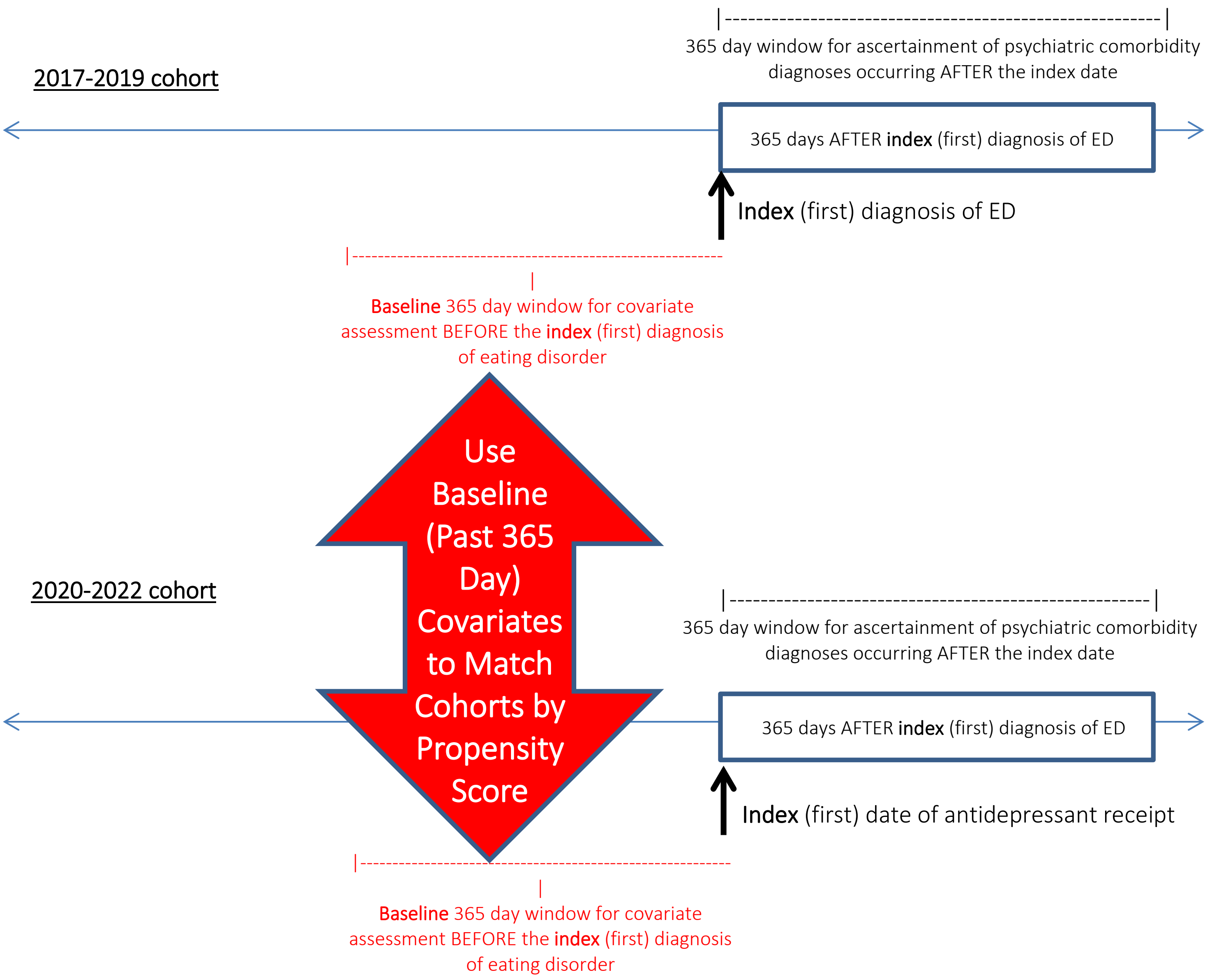

Supplement: Supplementary Information — We conducted propensity score-matched analyses that evaluate the risk of psychiatric diagnoses in the 365 days AFTER the index ED diagnosis. To accomplish this, people with EDs (above) and peers without EDs (below) are MATCHED with demographics and prior history of psychiatric diagnoses in the 365 days preceding the index date. [file Image1.pdf]
